# PANoptosis: Mechanism and Role in Pulmonary Diseases

**DOI:** 10.3390/ijms242015343

**Published:** 2023-10-19

**Authors:** Shiyi Chen, Jiacheng Jiang, Tongfu Li, Longshuang Huang

**Affiliations:** Shanghai Frontiers Science Center of Drug Target Identification and Delivery, School of Pharmacy, Shanghai Jiao Tong University, Shanghai 200240, China; syyy0741@sjtu.edu.cn (S.C.); cc0320@sjtu.edu.cn (J.J.); ltf-pharm2022@sjtu.edu.cn (T.L.)

**Keywords:** PANoptosis, pulmonary disease, pyroptosis, apoptosis, necroptosis

## Abstract

PANoptosis is a newly defined programmed cell death (PCD) triggered by a series of stimuli, and it engages three well-learned PCD forms (pyroptosis, apoptosis, necroptosis) concomitantly. Normally, cell death is recognized as a strategy to eliminate unnecessary cells, inhibit the proliferation of invaded pathogens and maintain homeostasis; however, vigorous cell death can cause excessive inflammation and tissue damage. Acute lung injury (ALI) and chronic obstructive pulmonary syndrome (COPD) exacerbation is related to several pathogens (e.g., influenza A virus, SARS-CoV-2) known to cause PANoptosis. An understanding of the mechanism and specific regulators may help to address the pathological systems of these diseases. This review presents our understanding of the potential mechanism of PANoptosis and the role of PANoptosis in different pulmonary diseases.

## 1. Introduction

Cell death was considered as an uncontrollable process (which is now named accidental cell death, ACD) until different morphological features of dying cells were observed by Karl Vogt in 1842. This unique type of cell death was finally termed apoptosis by John Kerr in 1972 [1]; since then, research on programmed cell death (PCD) has proliferated. In contrast to ACD, PCD involves governable biochemical cascades and occurs when cells confront intra/intercellular stimuli to maintain the homeostasis of the system. Besides apoptosis, several new types of PCD have been discovered over the past few decades and classified following the guidelines formulated by the Nomenclature Committee on Cell Death (NCCD), including necroptosis, ferroptosis, pyroptosis, parthanatos, etc. [2]. PANoptosis is a newly defined PCD, which was first proposed in 2019, involving key molecules of pyroptosis (P), apoptosis (A), and necroptosis (N) simultaneously [3]. Similar to other forms, it can be irritated by diverse triggers such as viruses, bacteria, and cytokines [4,5,6]. In contrast to apoptosis, PANoptosis is a type of inflammatory process like pyroptosis and necroptosis, resulting in cell membrane disruption and the release of cytokines and damage-associated molecular patterns (DAMPs) [6,7,8]. Normally, moderate PANoptosis is protective in defense against pathogenic microorganisms, whereas excessive cell death aggravates tissue damage ultimately. Pulmonary diseases are some of the most common health issues in the world, and inflammation provoked by pathogens, allergens, and cigarette plays an important role in the progression of the disease. There is increasing evidence that PANoptosis is implicated in the pathogenesis of pulmonary diseases [6,7,8,9].

In this review, we discuss the molecular mechanisms underlying the activation of PANoptosis and provide an overview of PANoptosis in pulmonary diseases. We then focus on the possibility of regulating PANoptosis as a therapeutic strategy and the potential targets for intervention.

## 2. General Crosstalk between Apoptosis, Pyroptosis, and Necroptosis

**Figure 1:**
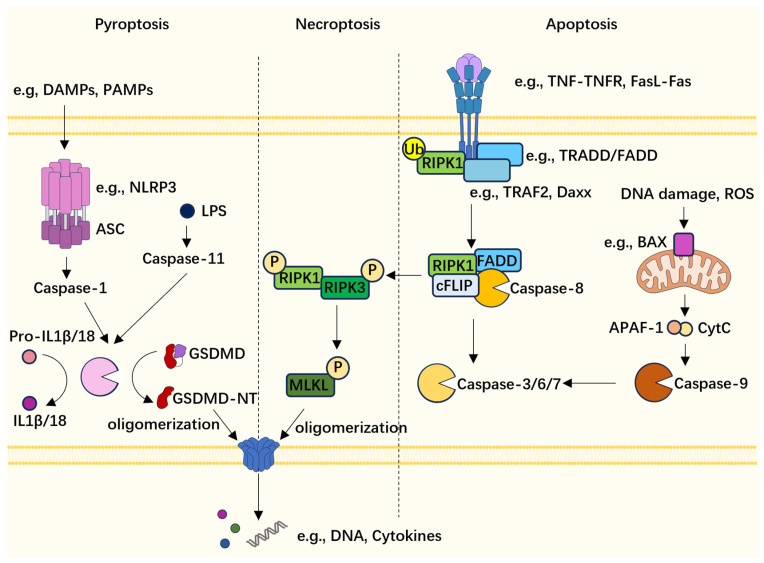
Molecular mechanism of pyroptosis, apoptosis, and necroptosis. Canonical pyroptosis can be triggered by diverse stimuli, such as pathogen-associated molecular patterns (PAMPs) and DAMPs, through which a specific sensor is activated, followed by the recruitment of the adaptor protein ASC and pro-caspase-1. Pro-caspase-1 activates itself via auto-cleavage, hence converting the pro-IL-1β and IL-18 into their mature forms and GSDMD into the pore-forming N-terminal. Non-canonical pyroptosis is based on the cytosolic LPS-induced activation of murine caspase-11 or human caspase-4/5, which also cleaves GSDMD and further activates NLRP3 (K^+^ efflux). Intrinsic apoptosis is mediated by the oligomerization of BAX or BAK on the mitochondrial membrane, and the pores allow cyt. *c* the enter cytoplasm and interact with APAF-1 to induce the autocleavage of pro-caspase-9, which leads to the following apoptosis cascade. Extrinsic apoptosis is initiated by membrane receptors (e.g., TNFR, Fas–ligand interaction), and, immediately after this, TRADD/FADD and receptor-interacting serine-threonine protein kinase 1 (RIPK1) is recruited. When the deubiquitination of RIPK1 happens, apoptosis continues. Abundant cellular FLICE-inhibitory protein (cFLIP) in cytosol can inhibit caspase-8 activity, while the cellular stress hampers the inhibitory activity of cFLIP and permits a caspase-8-induced apoptosis cascade. However, in conditions in which caspase-8 is prohibited, RIPK1 recruits and phosphorylates RIPK3, and RIPK1/RIPK3 can further activate MLKL through phosphorylation, which confers upon MLKL the pore-forming activity. Finally, the MLKL pores on the cell membrane can lead to cell necroptosis.

Apoptosis, pyroptosis, and necroptosis are three ubiquitous PCD types characterized by distinct molecular mechanisms (Figure 1). Apoptosis is further divided into the extrinsic and intrinsic types. In the extrinsic apoptosis pathway, membrane receptors, especially the death receptor (e.g., TNFR, Fas), which contains the death domain (DD), recognize its specific ligands, including the cytosolic adaptor protein, which will be recruited to the receptor and bind it through DD [10,11]. These adaptors can bind and interact with pro-caspase-8 through the death effector domain (DED), forming the death-inducing-signaling complex (DISC), followed by the activation and auto-cleavage of caspase-8 [12]. The activated initiator caspase-8 then cleaves the executor caspase-3 and pro-apoptotic protein Bid, entering the final phase of apoptosis. Meanwhile, the intrinsic apoptotic pathway is triggered by non-receptor stimuli such as DNA damage, metabolic stress, and redox imbalance and involves mitochondrial outer membrane permeabilization (MOMP), which is mediated by pro-apoptotic Bcl2 family members (BAX, BAK, Bid, etc.) [13]. The loss of integrity of the mitochondrial membrane allows the release of cytochrome *c* (cyt. *c*) and other mitochondrial proteins, which subsequently interact with apoptotic protease activating factor-1 (APAF-1) to form the apoptosome and activate initiator caspase-9, which possesses a similar function in the apoptosis process to caspase-8 [14,15].

Pyroptosis is also divided into two major types: the canonical and non-canonical pathways. The supramolecular complex (inflammasome) consists of a sensor protein, adaptor, and caspase-1 and is involved in canonical pyroptosis [16]. The sensor proteins mainly include Nod-like receptors (e.g., NLRP1, NLRP3, NLRP6), absent in melanoma 2 (AIM2), and MEFV innate immunity regulator (Pyrin) [17]. These sensor proteins can be activated by various pathogen-associated molecular patterns (PAMPs) and DAMPs and recruit the adaptor protein apoptosis-associated speck-like protein containing CARD (ASC) and bind it through the pyrin domain (except NLRC4, NLRP1b, etc.) [18,19]. The caspase activation and recruitment domain (CARD) of ASC and some sensors can directly interact with the CARD domain of pro-caspase-1, conferring proteolytic activity upon caspase-1 [20]. Non-canonical pyroptosis happens when human caspase-4/5 or murine caspase-11 perceives the intracellular liposaccharide (LPS) [21]. Moreover, reactive oxygen species (ROS) and IFN-β regulate the expression and activation of caspase-11 [22,23]. The activation of caspase proteins finally leads to the maturation of inflammatory cytokines IL1β and IL18, the cleavage of pore-forming protein gasdermin D (GSDMD), and cell death [24]. Moreover, ninjurin 1 (NINJ1) is recruited to facilitate the disruption of the plasma membrane [25].

Necroptosis is a type of PCD that shares similar morphological features with necrosis and is part of the signaling pathway with apoptosis [26]. In extrinsic apoptosis, death receptors match with the ligands and promote the formation of DISC, where caspase-8 activates and cleaves receptor-interacting serine-threonine protein kinase 1 (RIPK1), while, in necroptosis, caspase-8 is inhibited by cellular FLICE-inhibitory protein (cFLIP) and RIPK1 binds RIPK3 through homotypic interaction motifs (receptor-interacting protein kinase homotypic interaction motifs, RHIMs) to form a necrosome [27,28]. In the necrosome, auto- and cross-phosphorylation occur to activate RIPK1 and receptor-interacting serine-threonine protein kinase 3 (RIPK3), and it subsequently recruits and phosphorylates mixed-lineage kinase domain-like protein (MLKL), which could also form membrane pores through oligomerization and lead to plasma membrane rupture [29]. Furthermore, pathogen recognition receptors (PRRs), including Toll-like receptors (TLR3, TLR4) and Z-DNA binding protein 1 (ZBP1/DAI/DLM-1), could activate the catalytic function of RIPK3 in an RIPK1-independent manner and cause cell necroptosis [30,31,32].

Between apoptosis and necroptosis, there is an innate connection regulated by caspase-8, and mounting evidence shows interplays among the three cell death forms. Potassium efflux is a common trigger for NLRP3, and activated MLKL can lead to a drop in intracellular potassium levels to activate NLRP3 as the MLKL pore on the cell membrane allows the efflux of K^+^ [33,34]. For apoptosis and pyroptosis, the caspase family proteins are important in the crosstalk. Caspase-8, for example, can directly cleave GSDMD in Yersinia infection when transforming growth factor-β-activated kinase 1 (TAK1) is inhibited by YopJ, permitting the activation of caspase-8 [35,36]. Caspase-3, the executor in apoptosis, can activate gasdermin E (GSDME/DFNA5) in cancer cells receiving chemotherapy; in contrast, it inactivates GSDMD by cleaving it at D88 sites (destroying the N-terminal pore formation structure) [37,38,39]. Moreover, in dietary antigen-exposed intestine epithelial cells, the p13 fragment (the N-terminal cleaved at D88) was found to enter the nucleus and facilitate STAT1, which regulates the expression of MHC-II [40]. When RANK, another TNFR family member, is activated by its ligand RANKL, caspase-8 and caspase-3 cleave GSDMD together to produce a non-lytic p20 fragment and modulate lysosome function [41]. Besides the apoptotic caspase participating in the cleavage of GSDMD, caspase-1 cuts Bid at D59 to potentiate apoptosis [42,43]. Furthermore, GSDMD pores are found on the mitochondrial membrane and allow the release of mitochondrial ROS, mtDNA, and proteins such as cyt. *c*, to trigger the apoptosis pathway and regulate the innate immune responses [44,45].

## 3. Components and Assembly of PANoptosome

**Figure 2:**
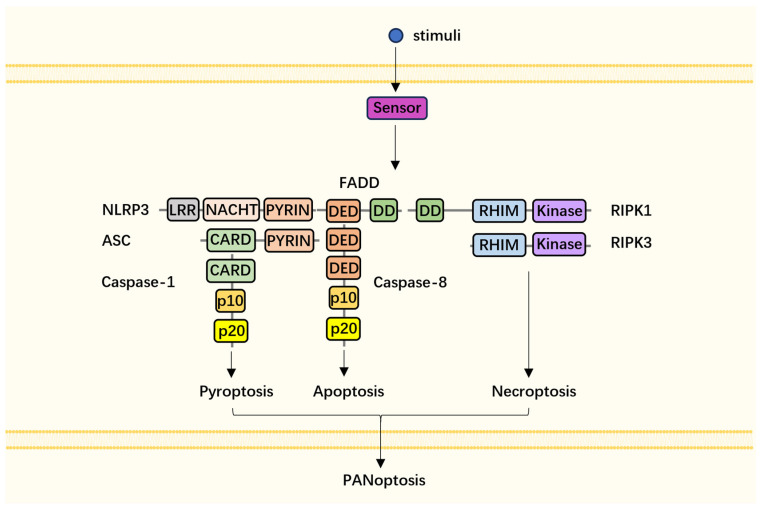
Components of PANoptosome. Various stimuli, including SARS-CoV-2, HSV-1, and TAK inhibitors (e.g., YopJ), can activate intracellular sensors of PANoptosis, such as ZBP1, AIM2, and RIPK3. The activated sensor then initiates the reassembly of other components of the huge multi-protein complex, the PANoptosome. Proteins involved in the PANoptosome interact through their homotypic (RHIM-RHIM, DD-DD, DED-DED, CARD-CARD) or heterotypic (DED-PYRIN) domains and finally trigger three independent cell death signals and result in cell PANoptosis.

As mentioned previously, pyroptosis, apoptosis, and necroptosis happen concurrently in PANoptosis and the interaction among them makes this process achievable. Similar to the inflammasome, apoptosome, and necrosome, a large multicomplex (PANoptosome) assembled through homotypic (RHIM-RHIM, DD-DD, DED-DED, CARD-CARD) and heterotypic (DED-PYRIN) domain interactions between proteins provides the platform for PANoptosis. The complex is composed of three parts: (1) a sensor for PAMPs or DAMPs, such as ZBP1, AIM2, or RIPK1 adaptors, to link sensors and effectors, including ASC and Fas-associated via death domain (FADD); (2) a catalytic effector like caspase-1, caspase-8, RIPK1, or RIPK3 (Figure 2 [46]).

ZBP1 is a cytosolic Z-nucleic acid sensor that contains the RHIM domain, through which it can bind with RIPK3 to induce cell death, and the Zα domain, which detects Z-nucleic acid [4,47]. The ZBP1-PANoptosome was first observed in the influenza A virus (IAV) infection. IAV is one of the negative-sense single-stranded RNA viruses, and ZBP1 can recognize the ds-RNA produced during the replication of the IAV genome in the host cell with the Zα2 domain. After this, ZBP1 interacts with RIPK3 and triggers cell necroptosis, as previous research has shown. However, the simple knockout of MLKL cannot entirely save the BMDMs infected with IAV, which implies that another type of cell death happens at the same time or as compensation [48]. In apoptosis, RIPK3 can interact with RIPK1 via the RHIM domain, and RIPK1 recruits caspase-8 through adaptor protein FADD to initiate cell death [1,4]. The same process occurs in IAV infection, and the knockdown of both RIPK3 and caspase-8 can effectively rescue the cell death induced by IAV [48]. In the previous study, IAV also induced NLRP3 inflammasome activation and pyroptosis [49,50]. Moreover, in ZBP1 or RIPK3 knockdown cells, IAV-induced caspase-1 and GSDMD cleavage can also be hampered, supporting the notion that ZBP1 may regulate NLRP3 in this situation [48]. Furthermore, co-immunoprecipitation (Co-IP) and immunofluorescence (IF) showed the co-localization and interaction among ZBP1, RIPK3, RIPK1, FADD, caspase-8, ASC, and caspase-1, indicating the existence of a multiprotein complex [48]. ZBP1 is found to interact with TANK-binding kinase 1 (TBK1) and interferon regulatory factor 3 (IRF3) to regulate IFNβ production when detecting DNA viruses (e.g., herpes simplex virus 1 (HSV-1)) or mitochondrial DNA, while, in the IAV infection model, ZBP1 activation relies on the IFNβ-IFNAR-STAT1/STAT2/IRF9 (interferon-stimulated gene factor 3 (ISGF3) complex) pathway, suggesting that IAV activates other RNA virus sensors, priming for the formation of the ZBP1-PANoptosome [51,52]. Besides this, caspase-6 is also a component of the ZBP1-PANoptosome and can bind with RIPK3 to enhance the interaction between RIPK3 and ZBP1 [53].

Similar to ZBP1, AIM2 is also a sensor for cytosolic DNA, and researchers show that HSV-1 and *Francisella novicida* (*F. novicida*) can induce PANoptosis through AIM2. HSV-1 is a double-stranded DNA virus that has a short replication cycle (4-12 h) and leads to host cell death rapidly. *F. novicida* is a Gram-negative bacterium, and it can invade cells and trigger the type I IFN response through another cytosolic DNA sensor, cyclic GMP-AMP synthase (cGAS) [54]. The IFN expressed then matches with its ligand by autocrine and promotes the expression of guanylate-binding proteins, namely guanylate-binding protein (GBP)-2 and GBP-5, which can embellish bacteria and immunity-related GTPase B10 (IRGB10) that target the bacterial cell membrane and facilitate the release of bacterial DNA [55,56,57]. Therefore, AIM2 can recognize the released bacterial dsDNA and form the AIM2 inflammasome, which triggers pyroptotic cell death. However, the knockdown of ZBP1 and another inflammasome, Pyrin, can partially inhibit cell death and cytokine release in HSV-1 and *F. novicida* infection, demonstrating that ZBP1 and Pyrin cooperate with AIM2 to regulate cell death [9]. Moreover, the expression of ZBP1 and Pyrin is inhibited in AIM2 knockdown cells, which indicates that AIM2 acts upstream of ZBP1 and Pyrin in HSV-1 and *F. novicida* infection [9]. Moreover, Co-IP and IF showed that caspase-1, caspase-8, RIPK1, RIPK3, FADD, and ASC work together with AIM2, ZBP1, and Pyrin to construct the AIM2-PANoptosome [9].

When TNFα binds to TNFR, RIPK1 is recruited to the TNFR complex and decorated with a K63 polyubiquitin chain by E3 ligases, which allow the TABs-induced TAK1 activation and phosphorylation of RIPK1 by TAK1, and hence RIPK1-induced cell apoptosis is inhibited and nuclear factor kappa-B (NF-κB) and mitogen-activated protein kinase (MAPK) signaling are activated [58,59]. However, in apoptosis, the ubiquitination of RIPK1 can be reversed by cylindromatosis (CYLD), hence excluding the intervention of TAK1, and triggers cell death through caspase-8 independently of the kinase activity of RIPK1 [58,59]. There is no doubt that TAK1 is a key regulator of RIPK1 activity [58,59,60,61]. When using *Yersinia pseudotuberculosis* (Yp), whose effector protein YopJ can inhibit TAK1, pyroptosis, apoptosis, and necroptosis occur concomitantly, while, in RIPK1 knockdown cells, only violent necroptosis occurs [5]. This indicates that RIPK1 is a hub protein in Yersinia-induced cell PANoptosis and TAK1 is the sluice controlling RIPK1. In the RIPK1-PANoptosome, caspase-8, RIPK3, and NLRP3 are involved, as well as in the ZBP1-PANoptosome [5,35,62,63].

## 4. PANoptosome in Pulmonary Disease

### 4.1. Acute Lung Injury (ALI)/Acute Respiratory Distress Syndrome (ARDS)

ALI/ARDS is associated with uncontrolled lung inflammation, characterized by cell death, the loss of cell–cell connections, and neutrophil infiltration, and results in tissue edema and acute respiratory failure. A plethora of insults can cause acute lung damage, including infection, systemic inflammation (e.g., systemic lupus erythematosus (SLE), rheumatoid arthritis (RA), sepsis), and trauma.

Pathogens can trigger various biological processes in the host, including cell death and innate immune activation. The types of cell death are related to the pathogen burden, and apoptosis often occurs when the burden is relatively low, while the excessive proliferation of pathogens may lead to cell lysis. The death of cells is somewhat protective as they release “alarming” molecules for the immune system, and, in contrast, it also facilitates the dissemination of pathogens and exacerbation of inflammation, as PANoptosis happens during pathogen infection. Severe influenza virus infection is manifested as ARDS, among which IAV is the most virulent one [64,65]. The virus activates TLRs and retinoic acid-inducible gene I (RIG-I) through ssRNA or dsRNA (during replication) and initiates the transcription of anti-virus cytokine IFNs mediated by IRF3/7 and mediates the antiviral immune process [48,66]. However, the IFNs exert adverse effects and can cause immunopathology in acute viral infection [67,68]. As mentioned previously, in IAV infection, IFNs induce the expression of ZBP1, which can recognize the Z-RNA during virus proliferation via the Zα domain and regulate the following cell PANoptosis (including epithelial cells, macrophages, endothelial cells) and deteriorate the immune response and tissue microenvironment [48]. A similar process is seen in another single-strain RNA virus, SARS-CoV-2, which was responsible for the global pandemic that began in 2019. Research also shows that PANoptosis can not only be triggered by the Z-RNA from SARS-CoV-2, but also the cytokine storm (mainly triggered by TNFα and IFN and followed by the activation of the STAT1-IRF1-iNOS-NO axis) that occurs during infection. IAV infection can also induce a cytokine storm and further exacerbate the inflammation of the lungs [6,7,48,69]. The difference is that the knockout of ZBP1 could rescue SARS-CoV-2-infected mice but not IAV-infected ones, with no reduction in virus titers; this may partially be attributed to the nonstructural protein 1 (NS1) and 2 (NS2) of IAV, which can inhibit the production of IFNβ by interfering with RIG-I and the dimer of IRF7, respectively. This eventually hampers the IFNβ-induced antivirus response (e.g., the expression of antiviral proteins) [70,71,72]. Besides macrophages, T cells participating in the adaptive immune phase of virus infection are also found to experience cell death (including NLRP3-mediated pyroptosis) during HIV infection [73]. The death of CD4+ T cells can be caused by reverse transcription intermediates from intruded HIV, and the release of IL-1β is partially inhibited by caspase-8 inhibitors and further inhibited by pan-caspase inhibitors, indicating that caspase-8 interferes with the activation of pyroptosis in this process, proposing that the HIV-induced PANoptosis may also occur in CD4+ T cells [74,75].

Apart from viruses, bacteria, fungi, and parasites also lead to ARDS, which often happens in the early phase of pneumonia, sepsis, or severe trauma [76]. The inhalation-acquired infection of *F. novicida* and *Yersinia pestis* (*Y. pestis*), ingestion-acquired infection of *Salmonella Typhimurium* (*S. Typhimurium*) and *listeria monocytogenes* (*L. monocytogenes*), and post-viral infection of a conditional pathogen such as *Staphylococcus aureus* (*S. aureus*) can cause ARDS to varying extents [77,78,79]. As mentioned previously, *Y. pestis* activates the RIPK1-PANoptosomeb by inhibiting the activity of TAK1; *F. novicida* triggers the AIM2-PANoptosome through type I IFN, but the type of PANoptosome and the way in which it is triggered by other pathogens remain unclear. When pathogens invade the host, they can choose to reside within or outside the host cells, but, regardless of how they survive, they are ultimately “eaten” by phagocytes such as macrophages and neutrophils. After the elimination of pathogens, the phagosome will detach from the plasma membrane and fuse with other cellular structures like the lysosome in macrophages or pre-packaged storage azurophil granules in neutrophils [80]. The content in the newly formed compartment is digested by different enzymes, including hydrolytic enzymes, elastase, etc., and some are processed into antigens for presentation, while some are released into the cytoplasm in their detectable forms (e.g., dsDNA), and others remain in the compartment [81,82]. The released bacterial dsDNA can be detected by the cGAS, a cytosolic DNA sensor that then activates the stimulator of interferon genes (STING)-TBK1-IRF3 signaling pathway [83]. Research reports that the STING natural agonist cyclic GMP-AMP (cGAMP) and synthetic non-cyclic dinucleotide diABZI can induce PANoptosis and neutrophilic lung inflammation, and the referenced cGAMP activates the NLRP3 inflammasome [8]. This suggests that the bacterial DNA-induced expression of IFNβ, together with other pro-inflammatory cytokines like TNFα, activates ZBP1 and RIPK1/3 to mediate PANoptosis, and this is different from the PANoptosis caused by the SARS-CoV-2-induced cytokine storm, which is believed to be dependent on the STAT1-IRF1-iNOS-NO axis [6,8]. The DNA from neutrophil extracellular traps (NETs) also plays an important role in this process, and adding DNase I to digest the extracellular DNA can effectively reverse the inflammatory-related tissue damage [8].

In SLE, the type I IFN reaction is non-negligible, and self-DNA—for instance, mitochondrial DNA (mtDNA)—is one of the triggers of the process. A study shows that mitochondria of red blood cells (RBCs) in SLE patients are mistakenly retained, and these RBCs are prone to opsonization by anti-RBC antibodies, which mediate the FcγR-dependent phagocytosis of macrophages [84]. Similar to pathogens, the immune complex (IC, RBC, and anti-RBC) is digested and mtDNA is released into the cytosol and promotes the production of IFN [84]. The self-DNA-induced inflammasome activation and release of IL1β (pyroptosis) depend on the presence of autoantibodies, further indicating that self-DNA activates PCD in SLE via its cytosolic form [85]. Besides this, the self-DNA antibodies can interact with TNFR1 and lead to fibroblast apoptosis or necroptosis, and the increased expression of MLKL is also seen in PBMCs from SLE patients [86,87]. The mtDNA can change from the usual B-DNA into Z-DNA form with the help of a specific DNA topoisomerase and its inherent torsional stress, and it is subsequently detected by the cytosolic Z-NA sensor ZBP1, which can form the ZBP1-PANoptosome under different conditions and is IFN-inducible [88]. Thus, self-mtDNA may trigger multi-tissue PANoptosis in SLE.

### 4.2. Asthma

Asthma is a chronic inflammatory lung disease that affects people of all ages, and the symptoms include a persistent cough, repeated wheezing, breathlessness, and chest tightness because of the narrowing of the airways and the excess production of mucus [89]. Environmental allergens including dust mites, fungi, and pollens can trigger T2-type inflammation-dominated allergic asthma, in which epithelium damage and immune cell injury are universal [89]. As the first barrier and sensor for allergens, epithelial cells are directly targeted, and the invasion of pathogens leads to increased oxidative stress and subsequent cell apoptosis, which is also observed in asthmatic children [90,91]. Toluene diisocyanate (TDI) is a classical trigger of allergic asthma, and research suggests that it can induce bronchial epithelial cell pyroptosis in an NLRP3-dependent way and exacerbate airway inflammation. GSDMB, another member of the gasdermin family, may also participate in the process [92,93]. In previous research, pyroptosis is usually connected with immune cells, and the macrophages in perfluoroalkyl substance (PFAS)-induced asthma experience pyroptosis mediated by AIM2 activation [94,95]. Eosinophils are essential in the development of asthma, and the damaged epithelial cells may cause the necroptosis of eosinophils in ovalbumin (OVA)/Complete Freund’s Adjuvant (CFA)-induced model mice; meanwhile, the deletion of MLKL on eosinophils can effectively alleviate airway hyperreactivity (AHR) [96]. Apoptosis, pyroptosis, and necroptosis are all observed in the process of asthma, but there is less evidence that they occur in the same types of cells simultaneously, and it is unclear whether allergens can trigger PANoptosis as pathogens do in ALR/ARDS. Moreover, although TNFα is elevated in allergic asthma, the expression of IFNs is usually impaired, and the TNFα plus IFNγ-induced PANoptosis may be limited in normal allergic asthma [97]. However, the situation is different with asthma exacerbation, whether bacteria including conditional pathogens from the lung microbiota or viruses infected after the onset of asthma can significantly promote IFNs and other pro-inflammatory cytokines, which upregulate the expression of the non-canonical caspase-11 inflammasome through the activation of STAT1 parallel to the ROS-JNK pathway directly triggered by pathogens [22,23,98]. Moreover, DAMPs from injured cells in other diseases also occur in asthma and asthma exacerbation, but the specific ones that can trigger PANoptosis, and whether they induce the same process in asthma, still need to be determined [99,100].

### 4.3. Idiopathic Pulmonary Fibrosis (IPF)

IPF is a chronic, progressive interstitial pneumonia, exhibiting cough and dyspnea (partially attributed to decreased lung volume and capacity), especially during exercise [101]. IPF enters into the acute exacerbation phase in the absence of infection, heart failure, and pulmonary embolism, resulting in rapid and devastating damage to lung tissue, and it is the leading cause of IPF-related death [101,102]. Alterations in oxidative stress and endoplasmic reticulum stress such as the unfolded protein response (UPR) are common in IPF and contribute to increasing epithelial cell apoptosis, characterized by the elevated cleavage of caspase-3 and oligomerization of Bax [103,104]. The master regulator of fibrosis, TGF-β, can not only stimulate the expression of proinflammatory and fibrogenic cytokines (e.g., TNFα, IL-1β, PDGF), but also mediate epithelial cell apoptosis through the upregulation of pro-apoptotic BCL2 family proteins, increasing oxidative stress and activating the Fas–caspase-8 signal [105,106]. Inflammasome activation is detected in pulmonary fibrosis as well, affecting epithelial–mesenchymal transition and epithelial cell pyroptosis [107,108,109]. The increment in the microbial burden and alteration of the microbial composition in IPF warn lung-resident immune cells and initiate the immune response and immune cell PCD [110,111]. Furthermore, the transcriptome sequencing results of the lungs of IPF mice showed that ZBP1, IFN-related genes such as ISG15, interferon-induced protein with tetratricopeptide repeats 1 (IFIT1), and IRF7 were significantly upregulated, indicating that IFN- and ZBP1-mediated PANoptosis may happen in IPF [112]. Neutrophils increased in the fibrotic lung tissue owing to CXCL1/2 and CXCL12 chemokines produced by lung cells in IPF mice, and the neutrophil elastase (NE) from NETs affected the proliferation and differentiation of fibroblasts [113,114,115,116]. In the STING agonist-induced cell PANoptosis in ARDS, the inhibition of NET formation can effectively alleviate cell death, but it remains unclear whether the self-dsDNA from NETs can induce a similar PANoptosis process in IPF progression [8].

### 4.4. Chronic Obstructive Pulmonary Syndrome (COPD)

COPD is a common airway disease, causing emphysema, inflammation, and abnormal mucus production, and has become the third leading cause of death worldwide [117]. Among the risk factors of COPD, smoking (including second-hand smoking and using electronic nicotine device systems) is the most frequent one, followed by exposure to other types of particles matter such as air pollutants, a previous lung infection, and lung cancer [117]. Abnormal cell death appears in COPD, triggering an inflammatory response, and is tightly linked with disease progression. Single-cell RNA sequencing (sc-RNA seq) shows that the expression of RIPK1, involved in both apoptosis and necroptosis, is elevated in various epithelial cells, alveolar macrophages (AMs), T cells, and B cells in the lungs of COPD patients [118]. Similarly, the total proteins of RIPK3 and MLKL, downstream of RIPK1, are also increased in the epithelial cells and macrophages of COPD patients, and the knockdown of RIPK1 or MLKL or the use of RIPK1 inhibitors could effectively reduce airway inflammation, remodeling, and emphysema in cigarette smoke extract (CSE)-induced COPD mice [118,119]. Other research showed that CSE caused apoptosis in both human and mouse epithelial cells and increased the expression of inflammatory cytokines such as nitric oxide (NO), IL-6, and TNFα, promoting the development of COPD. Endothelial cell apoptosis during COPD is also widely observed [120]. An analysis of the caspase-8 levels of COPD patients found that the caspase-8 level was associated with COPD development, indicating the role of apoptosis in COPD [121]. Moreover, caspase-8 is an important component of different PANoptosomes and also a hub regulator of different types of PCD, further proving the correlation between COPD and PCD. The canonical inflammasome pathway NLRP3/caspase-1/GSDMD is also blamed for the death of epithelial cells and AMs in COPD as ROS accumulate after the inhalation of CSE [122,123]. IFNγ is known to be elevated in COPD patients, and it is reported to enhance LPS-induced STAT1 activation in AM in COPD patients [124,125]. Furthermore, in virus infection-induced COPD exacerbation, the production of IFNβ is raised, although it is known to be deficient in COPD patients [126,127]. PANoptosis may play a unique role in infection-induced COPD exacerbation [6,7,120].

### 4.5. Lung Cancer

Resistance to cell death is a hallmark of cancer, and, in contrast to other diseases, PCD, including immunogenic cell death (ICD), and PANoptosis are important to reverse cancer in chemo-, immuno-, and radiotherapy [128,129]. A series of studies focused on the relationship between PANoptosis-related genes and the prognosis of lung, gastric, colon, and liver cancer, and it found that higher expression of these genes was correlated with longer disease-free survival (DFS), lower metastasis, and more immune cell infiltration, indicating the positive role of PANoptosis in disease prognosis and treatment [130,131,132,133,134,135,136]. The ZBP1-PANoptosome also participates in tumor regression, and this process is regulated by an RNA modification enzyme, adenosine deaminase acting on RNA 1 (ADAR1), which contains the homologous Zα domain, through which it can bind with ZBP1 directly and block the sensor of intracellular Z-NA [137,138,139]. A combination of IFN and nuclear transport inhibitors (KPT-330) to inhibit the translocation of ADAR1 from the nucleus to cytoplasm can potentiate tumor cell PANoptosis and suppress tumor progression [138]. The accumulation of superfluous intracellular ROS in nutrient and stress factor 1 (NSF1-aconitase 1 (ACO1)) or glycolysis (hexokinase)-inhibited tumor cells also results in PANoptosis, implying a potential regulator of PANoptosis [140,141]. Moreover, proteins like caspase-6/8 and IRF1, regulating PANoptosis in other diseases, can also regulate PANoptosis in cancer [142,143,144] (Table 1).

### 4.6. Other Pulmonary Diseases

Various types of cell death also occur in other pulmonary diseases, like pulmonary arterial hypertension (PAH) and tuberculosis [145,146,147]. A study shows that the expression of several inflammasomes, including NLRP3, NLRC4, and AIM2, and the necroptosis-associated RIPK3 and the apoptotic caspase-8, are elevated in monocrotaline-induced PAH [146,148]. The inhibition of caspase-8 or knockout of caspase-8 and RIPK3 simultaneously can alternatively affect the activation of caspase-1 and GSDMD in PAH mice, indicating the possibility of the assembly of the PANoptosome as caspase-8 is a key component of the multi-protein scaffold and an important regulator of PANoptosis, but more experiments including Co-IP are necessary to verify the existence of the PANoptosome in this process [148]. Mycobacterium tuberculosis (Mtb) is an intracellular bacterium that is responsible for tuberculosis, and phagocytes such as neutrophils and macrophages can prevent the proliferation and dissemination of Mtb by commencing non-lytic cell death (e.g., apoptosis) [149]. In contrast, the pathogen struggles to escape from the lysosome and activates the intracellular dsDNA sensor AIM2 with its genomic DNA to trigger cell pyroptosis and spread in the tissue [147,150]. Since Mtb shares several characteristics with F. novicida, as mentioned before (intracellular bacterium, produces dsDNA and activates expression IFNs and AIM2), PANoptosis mediated by AIM2 may be a potential strategy allowing it to escape from cells.

**Table 1:** Potential triggers of PANoptosis and associated diseases.

Involved PANoptosome Component	Trigger	Disease	Reference
ZBP1-PANoptosome	IAV, SARS-CoV-2, diABZI (STING agonist)	ALI/ARDS	[8,48,69]
RIPK1/3-FADD-caspase-8	SARS-CoV-2	ALI/ARDS	[6]
AIM2-PANoptosome	HSV-1, *F. novicida*	ALI/ARDS	[9]
RIPK1-PANoptosome	*Y. pestis*	ALI/ARDS	[5]
ZBP1	self-mtDNA	SLE	[81,82,85]
NLRP3	TDI	asthma	[89,90]
AIM2	PFAS	asthma	[91,92]
NLRP3	bleomycin	IPF	[104,105,106]
RIPK1, NLRP3	CSE	COPD	[115,116,119,120]
ZBP1-PANoptosome	ADAR1 knockdown or IFNs plus nuclear transport inhibitors KPT-330	cancer	[135,136]

Abbreviations: ZBP1, Z-DNA binding protein 1; IAV, influenza A virus; ALI, acute lung injury; ARDS, acute respiratory distress syndrome; RIPK1, receptor-interacting serine-threonine protein kinase 1; RIPK3, receptor-interacting serine-threonine protein kinase 3; FADD, Fas-associated via death domain; AIM2, absent in melanoma 2; HSV-1, herpes simplex virus 1; *F. novicida*, *Francisella novicida*; *Y. pestis*, *Yersinia pestis*; mtDNA, mitochondrial DNA; SLE, systemic lupus erythematosus; NLRP3, NLR family pyrin domain containing 3; TDI, toluene diisocyanate; PFAS, perfluoroalkyl substances; IPF, idiopathic pulmonary fibrosis; CSE, cigarette smoke extract; COPD, chronic obstructive pulmonary syndrome; ADAR1, adenosine deaminase acting on RNA 1; IFNs, interferons.

## 5. Potential Inhibitors of PANoptosome

As PANoptosis is proven to aggravate inflammation and tissue damage, targeting the molecules involved can be a potential treatment for the disease, and recent works have made some progress in discovering candidates for these molecules (Table 2).

GSDMD and GSDME are two key final executors of PANoptosis and can be activated by caspases from both pyroptosis and apoptosis pathways. Necrosulfonamide (NSA) has been discovered as a necroptosis inhibitor, and recent research found that it blocked pyroptotic cell death in both primary murine and immortalized human and murine macrophages through direct binding to Cys191 of GSDMD and inhibited GSDMD-N-terminal oligomerization [151]. LDC7559/2618 are inhibitors of NET formation, and a pulldown with LDC7599/2618 from cell lysates showed that GSDMD is a potential target, but the ways in which it interacts with GSDMD remain unknown [152]. The high-throughput biochemical screening of FDA-approved drugs led to the discovery of another GSDMD inhibitor, disulfiram, which binds to the Cys191 of GSDMD as well [153]. Dimethyl fumarate (DMF) is also reported to interact with GSDMD by covalent bonds and form S-(2-succinyl)-cysteine (Cys192 of mouse GSDMD) [154]. Moreover, the N protein of SARS-CoV-2 is found to bind with the linkage of the GSDMD N-terminal and C-terminal and inhibits GSDMD cleavage by caspase-1, based on which peptide analogs can be designed [155].

MLKL, another pore formation protein in PANoptosis, is a potential target to inhibit cell death. Compound 1 (an ATP mimetic also named GW806742X), targeting the nucleotide-binding site of the MLKL pseudokinase domain, suppresses the conformational change in MLKL, retards MLKL membrane translocation, and inhibits necroptosis [156]. Different from GW806742X, the necroptosis-blocking compound 1 (NBC1) does not interact with MLKL directly, and it covalently conjugates the cysteine of HSP70 to block its protein chaperone function, hence inhibiting MLKL polymerization [157]. NSA, mentioned as a GSDMD inhibitor, can also directly target the Cys86 of human MLKL and block its translocation to the cell membrane [158]. RIPK1/3 act upstream of MLKL and are important in regulating PANoptosis and inflammation. RIPK1 inhibitors such as GSK481/547/963, Nec-1, and RIPA-56, and the RIPK3 inhibitor GSK872, are verified to inhibit various inflammation-associated diseases, including COPD and ALI [118,159,160,161].

Caspase proteins, participating in pyroptotic and apoptotic cell death, are also regulators and executors of PANoptosis. Inhibitors of caspase are mainly divided into two types: peptide-based or peptidomimetic inhibitors and non-peptidic compounds. For instance, the pan-caspase inhibitor quinoline-Val-Asp-difluorophenoxymethylketone (qVD-OPh) is reported to inhibit COPD in MLKL knockdown mice [119]. Moreover, a widely used pan-caspase inhibitor, Z-VAD-FMK (ZVAD), can alleviate cytokine-storm-induced epithelial cell death combined with NSA [162].

**Table 2:** Potential targets in PANoptosis and their inhibitors.

Target	Compound	Potential Mechanism	Reference
GSDMD	Necrosulfonamide	binds to Cys191 of human GSDMD, inhibits GSDMD-NT oligomerization	[151]
	LDC7559/2618	unknown	[152]
	Disulfiram	binds to Cys191 of human GSDMD	[153]
	Dimethyl fumarate	binds to Cys192 of mouse GSDMD	[154]
MLKL	GW806742X	binds to nucleotide-binding site of the MLKL, suppresses the conformational change of MLKL, and retards MLKL membrane translocation	[156]
	NBC1	covalently conjugates cysteine of HSP70 to block its protein chaperone function and inhibits MLKL polymerization	[157]
	NSA	binds to Cys86 of human MLKL and blocks its translocation to the cell membrane	[158]
RIPK1	GSK481/547/963	inhibits Ser166 phosphorylation of RIPK1	[159]
	Nec-1	inhibits kinase domain of RIPK1	[161]
	RIPA-56	inhibits kinase domain of RIPK1	[159]
RIPK3	GSK872	binds to kinase domain of RIPK3	[160]
Caspase proteins	qVD-OPh	substrate analogue that binds to catalytic units	[119]
	Z-VAD-FMK	substrate analogue that binds to catalytic units	[162]

Abbreviations: GSDMD, gasdermin D; Cys, cysteine; MLKL, mixed-lineage kinase domain-like protein; HSP70, heat shock protein 70; RIPK1, receptor-interacting serine-threonine protein kinase 1; RIPK3, receptor-interacting serine-threonine protein kinase 3; Ser, serine.

The targets of PANoptosis are not confined to the executive proteins; adaptor proteins such as ASC and sensors like ZBP1 and AIM2 are also potential targets, but inhibitors of these proteins are limited. The disruption of protein–protein interaction is another potential strategy to inhibit the progression of PANoptosis, as various homotypic and heterotypic domain interactions take place to drive the process. Moreover, the liquid–liquid phase separation (LLPS)-mediated formation of membrane-less organelles was discovered in cGAS-STING, the NLRP6 inflammasome activation pathway, promoting specific immune signaling and becoming a potential target of cancer and infection [163,164,165]. Although the occurrence of LLPS in PANoptosis still needs to be verified, LLPS can be considered a target of PANoptosis as RIPK1–RIPK3 interaction can involve LLPS [166].

## 6. Conclusions

The crosstalk among different forms of cell death has promoted the discovery of PANoptosis, which is involved in the death of various cells under a diversity of conditions. In infection-induced ALI/ARDS, the assembly of the PANoptosome relies on the detection of pathogens by certain sensors, while the “sensor” of PANoptosis in other sterile inflammation-related diseases is still a mystery. It is necessary to verify whether and how PANoptosis is involved in the progression of asthma, IPF, and COPD. The role of other immune sensors, such as the NLRP1/6 inflammasome, cGAS, RIG-I, and TLRs, in PANoptosis also needs further exploration. Furthermore, it is important to find other key PANoptosis regulators and understand the mechanisms underlying them, to better control PANoptosis during disease.

## Data Availability

Not applicable.
